# Administration of Altrenogest to Maintain Pregnancy in Asian Elephants (*Elephas maximus*)

**DOI:** 10.3390/ani12141852

**Published:** 2022-07-21

**Authors:** Jack J. Kottwitz, Wendy Kiso, Dawn M. Boothe, Dennis Schmitt

**Affiliations:** 1 College of Veterinary Medicine, Michigan State University, East Lansing, MI 48824, USA; 2 College of Veterinary Medicine, Auburn University, Auburn, AL 46849, USA; boothdm@auburn.edu; 3 Ringling Bros. Center for Elephant Conservation, Polk City, FL 33868, USA; wkkiso@gmail.com (W.K.); dennisschmitt@missouristate.edu (D.S.); 4 White Oak Conservation, Yulee, FL 32097, USA; 5 William H. Darr College of Agriculture, Missouri State University, Springfield, MO 65897, USA

**Keywords:** altrenogest, asian elephant, miscarriage, progesterone, pregnancy

## Abstract

**Simple Summary:**

The Asian Elephant (*Elephas maximus*) is an IUCN (International Union of the Conservation of Nature) *Endangered* species that has interacted with humans for centuries. Despite advances in captive elephant breeding knowledge, worldwide populations continue to decline. Progesterone is a key reproductive hormone for the maintenance of pregnancy in mammalian species. The monitoring of serum progesterone levels has become a key method of management for captive breeding of elephants. The synthetic progesterone, altrenogest, has been administered to multiple species of mammals both for management of estrus and maintenance of pregnancy. This paper details three Asian elephant pregnancies maintained by the administration of altrenogest after endogenous progesterone levels decreased below the point needed to maintain pregnancy. Pharmacokinetic parameters of altrenogest administered orally as a single dose to nonpregnant pilot study elephants are presented as preliminary data on the administration of this drug to Asian elephants as a pharmacological means of maintaining pregnancy to term.

**Abstract:**

Progesterone and progesterone derivatives are key hormones in pregnancy maintenance in mammalian species. Cessation of pregnancy, including birth or miscarriage, is certain if levels of these hormones drop below a given species-specific requirement necessary to maintain pregnancy. The synthetic progestin, altrenogest, is FDA-approved in the United States for suppression of estrus or synchronization and is administered extra-label to multiple species to maintain pregnancies in cases of luteal deficiency or otherwise abnormally low progesterone levels. Three pregnant Asian elephants received altrenogest from 41 to 131 days during the final trimester of pregnancy, with parturition occurring from 15 to 31 days after altrenogest administration stopped. A single dose of 0.2 mg/kg altrenogest administered to two nonpregnant Asian elephants provided pilot pharmacokinetic data. Serum samples from two of the three clinical cases and the two pilot study elephants were analyzed using Ultra Performance Liquid chromatography coupled to a triple quadruple mass spectrometer (UPLC-MS). Small sample numbers limited analysis; however, the following were determined: AUC∞ of 635.4 ± 73.8 ng*h/mL, Cmax of 30.2 ± 14.4 ng/mL at a Tmax of 4 ± 2.8 h, terminal T1/2 of 47.5 ± 3.0 h, MRT of 36.0 + 3.4 h and Vd/F of 1243.8 + 275.0 L/kg. These data and the three described cases serve as an indication that altrenogest can be administered to Asian elephants as an exogenous progestin to support pregnancy in elephant pregnancies with low endogenous progestin levels.

## 1. Introduction

The reproductive status of Asian elephants (*Elephas maximus*), has been a concern for decades, if not centuries [1]. Multiple factors have contributed to the decline in the Asian elephant population, including habitat fragmentation, poaching driven by the illegal ivory trade, and the resultant pressures on remaining familial groups [2]. The ecological threats and concern for the species’ survival has resulted in managed populations being increasingly viewed as a component of species conservation, a source of vital reproductive research studies, and can serve as a potential source for the reintroduction of animals to the wild [1,2]. Stabilization and proliferation of captive elephant populations remain hampered by multiple challenges, ranging from logistical concerns such as fertility compatibility, physiological concerns such as bull sub/infertility, cow ovarian cycle abnormalities, uterine pathologies, gestational difficulties, and behavioral concerns such as mate incompatibility and poor libido that can limit managed breeding success [1,2,3].

Progesterone or its derivatives are essential hormones in mammals for the development and maintenance of uterine tissue to support and sustain the placenta and facilitate fetal growth [4,5]. The specific functions of progesterone and progestins are mediated by the progesterone receptor [4]. While these functions are not fully described in elephants, in other mammals’ uterine stromal cells respond to progesterone by producing paracrine factors that regulate the proliferation and/or differentiate functions of the uterine glandular and the endometrial luminal tissue [4]. A decrease or abnormal production of progestogens during pregnancy can cause delayed fetal development and ultimately result in miscarriage and fetal death [4,5]. Monitoring of elephant pregnancy via evaluation of progestins is accomplished via measurement of blood or urine progestins, with a sudden drop below 0.20 ng/mL and return to near zero (baseline) levels 2 to 3 days before parturition [1,6,7,8].

Altrenogest (allyltrenbolone, 17a-allyl-17b-hydroxy-estra-4,9,11-trien-3-one) is a synthetic progestogen labeled in the United States for administration to domestic horses and swine to suppress, control, and synchronize estrus in sexually mature females of each respective species [9,10]. Altrenogest is administered extra-label to both domestic horses and swine to maintain pregnancy [1,11,12,13,14,15,16].

The goal of this study was to describe the administration of altrenogest for the purpose of maintaining pregnancy in Asian elephants. The pharmacokinetics of altrenogest was determined in two nonpregnant Asian elephants (pilot study). Altrenogest was administered to maintain pregnancy in three Asian elephants due to a premature deficiency with endogenous progesterones returning to baseline levels before full-term gestation. Altrenogest concentrations were determined at varying times during therapeutic dosing in two pregnant Asian elephants. A third pregnant Asian elephant was also treated with altrenogest, but serum drug concentrations were not determined. The data gathered provide preliminary dosing information for this drug.

## 2. Materials and Methods

All participating elephants were housed at the same facility in Florida, USA. Altrenogest was administered by mouth, mixed with Gatorade^®^ (Pepsico Inc., Purchase, NY, USA) of the flavor preferred by the elephant (typically fruit punch), followed by the elephant’s normal diet for that time of day. Blood samples (10 mL) were collected from an ear vein utilizing standardized blood collection techniques for all samples from all elephants. All blood samples were centrifuged, the serum collected, divided into 2 mL aliquots, and frozen at −80 °C until time of analysis.

### 2.1. Pilot Study in Non-Pregnant, Non-Breeding Animals

A single oral dose (0.02 mg/kg) of altrenogest (Regu-Mate (altrenogest) Solution 0.22%, Merck & Co., Inc., Rahway, NJ, USA) was administered to two nonpregnant and non-breeding female elephants. Both elephants had previously had multiple pregnancies resulting in live calves. Samples (10 mL whole blood) were collected from those elephants using the same methods as for the pregnant elephants, at times: 0, 0.5, 1.0, 1.5, 2, 4, 6, 8, 24, 48, 72, and 96 h post drug administration.

### 2.2. Clinical Cases

Pregnant females were chosen to receive altrenogest supplementation based upon having an apparently healthy pregnancy confirmed via ultrasound, yet serum progesterone concentrations were decreasing towards or falling below baseline (<0.20 ng/mL) before 620 days of gestation. Altrenogest was administered to each pregnant elephant at a dose range of 0.01 to 0.02 mg/kg (44–88 mg/day) with single dosing occurring between 6:00 and 10:00 AM and BID drug administration occurring between 6:00 and 10:00 AM and 4:00 and 8:00 PM.

Blood was collected to evaluate serum progesterones in each case as part of routine pregnancy monitoring. Pregnant females underwent routine transabdominal or transrectal ultrasound examinations throughout their pregnancy to monitor fetal health and development [17,18,19]. Serum was analyzed for altrenogest from Case 1 and Case 2 utilizing banked serum samples collected during pregnancy for progesterone monitoring. During pregnancy, samples were collected twice per week for each case. Sample collection increased to once per day the week prior to stopping altrenogest therapy. Sample collection continued until the time of parturition for each case even though maternal serum progesterone concentrations were below baseline levels. All samples were stored at −80 ^o^C for a maximum of 3 years. Samples from clinical cases were transported frozen on dry ice for altrenogest analysis. Case 3 did not have serum analyzed to determine altrenogest concentrations. The number of samples analyzed from clinical cases and corresponding altrenogest doses administered are summarized in Table 1.

### 2.3. Case Descriptions

#### 2.3.1. Case 1

A 17-year-old nulliparous, 4055 kg cow was bred by artificial insemination for her second pregnancy (Table 2). Pregnancy was confirmed by ultrasonographic examination at 12 weeks of gestation. It was noted that maternal serum progesterone levels dropped below baseline (<0.20 ng/mL) at day 488 of gestation. Altrenogest was started at 44 mg (0.011 mg/kg) orally once per day on day 489 of gestation. Fetal movement was noted during routine ultrasound examination on day 491 of gestation. Cervical relaxation was noted during ultrasonographic examination on day 510 of pregnancy. The calf was noted as moving at that time, but maternal serum progesterones remained at baseline. Altrenogest administration was increased to 44 mg (0.011 mg/kg) orally twice per day on day 510 of gestation. Distinct fetal movements were not noted after day 523 of gestation. This dose was continued until 621 of gestation, at which time altrenogest administration was stopped. Active labor was noted on day 636 of gestation, 15 days after discontinuing altrenogest. A stillborn calf was delivered on day 636 of gestation.

#### 2.3.2. Case 2

A 48-year-old multiparous (seventh pregnancy), 4407 kg cow was bred through natural breeding (Table 2). Pregnancy was confirmed in this cow by prolonged elevated serum progesterone levels. Physical conformation prevented visualization of the uterus in this cow until day 358 of gestation, but fetal viability could not be determined. Maternal serum progesterone levels were noted as below 0.20 ng/mL on day 351 of gestation. As a result, altrenogest was started at, 88 mg (0.02 mg/kg) orally once per day. It was noted on day 358 of gestation that maternal serum progesterones had rebounded to a normal pregnancy level of 0.499 ng/mL, however, because of concern for fetal viability, the decision was made to continue altrenogest administration. A fetal heartbeat was observed during ultrasonographic examination on Day 363 of gestation, confirming fetal viability. An active and viable fetus identified by visualization of the fetal heartbeat was observed during weekly ultrasonographic examinations through day 446 of gestation, however maternal progesterones dropped to below baseline (<0.20 ng/mL) again at that time. The altrenogest dose was increased to 88 mg (0.02 mg/kg) by mouth twice per day on day 446 of gestation. Phenylbutazone therapy at a dose of 2 g (0.45 mg/kg) per day, orally in the AM was initiated at 547 days of gestation to manage lameness. This dose was increased to two grams in the AM and one gram in the PM (3 g total, 0.68 mg/kg per day), after two days of therapy and continued for twelve additional days. Ultrasonographic examination at day 468 of gestation confirmed calf viability, with visible calf movement and heartbeat. Altrenogest was discontinued on day 561 of pregnancy. An active calf was observed during ultrasonographic examination on Day 577 of gestation. During examination at 591 days of gestation, it was noted that the calves’ front feet had advanced into the birth canal, but active signs of labor from the dam were not identified. Oxytocin (60 IU, administered intramuscularly) was administered on day 592 of gestation, with the delivery of a stillborn calf occurring 36 min after oxytocin administration.

#### 2.3.3. Case 3

A 10-year-old primiparous, 2948 kg cow was naturally bred with pregnancy confirmed via transabdominal ultrasound 81 days post-breeding (Table 2). Routine transabdominal ultrasound confirmed fetal growth and development. At day 154 of gestation, elephant endotheliotropic herpesvirus (EEHV-5) was detected during herd screening in several individuals. There were no symptoms noted at this time in this cow and no treatment was initiated. Another young elephant in the same barn had been treated for EEHV-1A a few months prior, but EEHV-1 was never detected in this cow during bi-weekly routine testing. The pregnancy continued without any adverse health effects noted and observable fetal growth and movement were observable via transabdominal ultrasound. At 568 days of gestation, maternal serum progesterones fell below baseline (<0.20 ng/mL). Altrenogest administration began at a dose of 61.6 mg (0.021 mg/kg) orally twice per day. Altrenogest administration continued until day 621 of gestation. The calf was noted as active and the cervix of the dam was not dilated via transrectal ultrasound examination at the time of discontinuation of altrenogest. Daily ultrasonographic examination revealed an active, calf, with the relaxation of the cervix beginning to occur 5 days after discontinuing altrenogest. Milk was noted as being easily expressed from both mammary glands 7 days after discontinuing altrenogest on day 628 of gestation. Ultrasonographic examination on day 634 of gestation revealed an active calf. Initial signs of labor were noted on day 635 of gestation. Active labor was observed during day 636 of gestation, but calf progression ceased. A live calf was delivered at 636 days of gestation, 15 days after discontinuing altrenogest administration, but the calf required manual extraction for delivery. No heartbeat or respirations were noted immediately after delivery. CPR was initiated, and a strong heartbeat was established, but respirations were variable. Over the next several hours, the calf was observed having multiple seizures with an elevating body temperature, which led to euthanasia 6.8 h after delivery. It should be noted that this cow became pregnant again, with a normal second pregnancy, not requiring altrenogest therapy, and successfully delivered a live calf.

## 3. Sample Analysis

### 3.1. Serum Sample Analysis

Altrenogest was quantitated in serum samples using Ultra Performance Liquid chromatography coupled to a triple quadruple mass spectrometer (UPLC-MS). The method was a modification of the previously described [20,21]. The UPLC-MS system consisted of an Agilent 1290 system coupled with an Agilent 6460 Triple Quad Mass Spectrometer (Agilent Technologies, Santa Clara, CA, USA) equipped with an electrospray ionization (ESI) interface. Chromatographic separation was achieved with a Zorbax SB C8, 1.8 µm, 2.1 × 50 mm chromatographic column (Agilent Technologies, Santa Clara, CA, USA) [20]. The mobile phase consisted of 5 mM ammonium acetate buffer and acetonitrile (VWR^®^, Radnor, PA, USA) using a gradient from 40% acetonitrile to 80% in 2 min with a flow rate set to 0.5 mL/min using an Agilent Jetstream electrospray ionization (AJ ESI) source [20,21]. Nitrogen gas was used as the drying (10 L/min at 350 °C), nebulizing (45 psi), and the collision gas. The capillary voltage was set to 4000 V. The mass spectrometer was operated in the positive mode and mass transitions were monitored using Multiple Reaction Monitoring (MRM). For quantification purposes the ion transitions were monitored for altrenogest and trenbolone (Internal standard) respectively as follows:

The standard curve for altrenogest ranged from 3.9 to 1000 ng/mL. This range was created by fortifying Asian elephant serum with known amounts of altrenogest reference standard and trenbolone internal standard (Sigma-Aldrich, St. Louis, MO, USA) that were prepared in 50:50 (*v/v*) acetonitrile/distilled water and acetonitrile, respectively.

Briefly, in 100 µL elephant serum samples, standards (3.91, 7.81, 15.63, 31.25, 62.5, 125, 250, 500 and 1000 ng/mL) and quality controls (3.91, 7.81, 62.5 and 500 ng/mL) were added to 100 µL of Trenbolone (25 ng/mL in acetonitrile), vortexed for 30 s, then cold acetonitrile (300 µL) was added, vortexed for 45 s and centrifuged at 14,000× *g* for 15 min. A portion of the supernatant (300 µL) was transferred to a clean glass tube, then dried under a nitrogen stream at 45 °C [20] The residue was reconstituted with 60 µL acetonitrile, vortexed, and 1 µL of the final solution was injected into the UPLC-MS System. The standard curves were accepted if the coefficient of determination (r2) was at least 0.999 and the predicted concentrations were within ±20% of the actual concentrations [20].

The UPLC-MS assay was validated for elephant serum. The linear correlation coefficient (r^2^) for altrenogest in elephant serum was 0.999, and the limit of detection (LOD) was 3.91 ng/mL. The lower limit of quantification (LOQ) for altrenogest in Asian elephant serum was 3.91 ng/mL. The upper limit of quantification (LOQ) for altrenogest in Asian elephant serum was 1000 ng/mL. The Accuracy (RSD%) for altrenogest in Asian elephant serum at 3.91, 7.81, 62.5, and 500 ng/mL was 8.0%, 6.5%, 3.2%, and 4.8% respectively. The Precision (RSD%) for altrenogest in Asian elephant serum at 3.91, 7.81, 62.5, and 500 ng/mL was 11.2%, 5.6%, 5.3%, and 3.6% respectively. The Percent Recovery for altrenogest in Asian elephant serum at 3.91, 7.81, 62.5, and 500 ng/mL was 108.6%, 107.9%, 107.9%, and 103.7% respectively.

### 3.2. Pharmacokinetic Analysis

#### 3.2.1. Pilot Study

Serum altrenogest concentration versus time data in the two nonpregnant elephants was subjected to non-compartmental analysis using the software program WinNonLin^®®^ (Phoenix WinNonLin Version 7.1; Cetara, Princeton, NJ 08540, USA). The area under the curve to infinity (AUC_∞_) was determined using the log-linear trapezoidal method. The actual maximum concentration (C_max_) occurring at time to maximum concentration (T_max_) was calculated. The slope of the terminal component of the drug elimination time curve was based on non-linear regression. Because altrenogest was not administered intravenously, the terminal component could not be confirmed to be elimination; therefore, both the elimination rate constant (K_el_) and half-life (T½) were reported as disappearance K_el_ and disappearance T_½_. Clearance (Cl) and volume of distribution (V_d_) were estimated because altrenogest was administered orally, not intravenously. Other parameters included mean residence time (MRT) and the percent of the AUC that was extrapolated from the terminal component of the curve. Pharmacokinetic parameters were reported as mean ± standard deviation with 95% confidence intervals.

#### 3.2.2. Pregnant Animals

Sample collection was not sufficiently consistent across time to allow modeling of the data.

## 4. Results

### 4.1. Pilot Study Elephants

**Figure 1:**
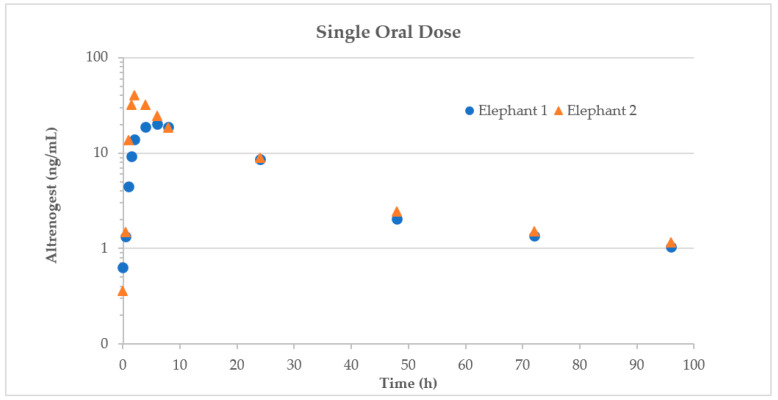
Time versus serum altrenogest concentrations in a pilot study of Asian elephants (*n* = 2) after a single oral dose of 0.02 mg/kg.

The maximum concentration (Cmax) of altrenogest after a single oral dose was 26.10 ± 6.07 ng/mL at a time to maximum concentration (Tmax) of 5 ± 1.0 h. (Figure 1) The terminal half-life (terminal T_½)_ was 47.54 ± 2.97 h with an elimination rate constant (K_el_ of 0.01 ± 0.001·1/h. The area under the curve to infinity (AUC∞) was determined to be 658.52 ± 45.89 h·ng/mL. The Mean Residence Time (MRT) was determined to be 5 ± 1 h. These parameters are summarized in Table 3.

### 4.2. Clinical Cases

**Figure 2:**
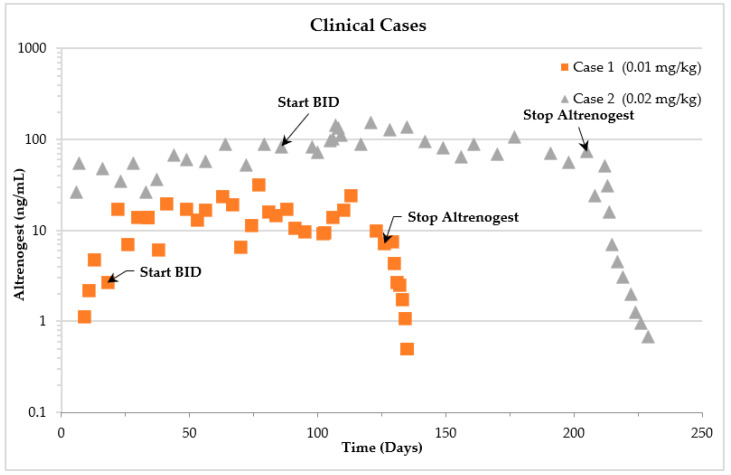
Time versus serum altrenogest concentrations in two pregnant Asian elephants after multiple dosing once per day or twice per day (BID) at 0.01 mg/kg (Case 1) or 0.02 mg/kg (Case 2).

The mean serum concentration for Case 1 while receiving a dose of 0.01 mg/kg once per day was 2.7 ± 1.5 ng/mL. When the dose was increased to twice per day, the serum concentration increased to 12.97 ± 6.9 ng/mL. Case 2 had a mean serum concentration of 51.0 ± 20.7 ng/mL while receiving a dose of 0.02 mg/kg once per day. The mean serum concentration increased to 86.2 ± 33.9 ng/mL when the dose frequency was increased to twice per day. These parameters are summarized in Table 1 and time vs. altrenogest concentration is depicted in Figure 2.

## 5. Discussion

This study represents pilot data for the pharmacokinetics of altrenogest administered to managed Asian elephants for use in future studies. This study serves to begin to establish both dosing and dosing interval recommendations for the administration of altrenogest to pregnant elephants to maintain pregnancy in the face of decreased progesterones which would otherwise result in pregnancy loss. Although this study demonstrated altrenogest can be administered and measured in Asian elephants, the biggest shortcoming of this study is the limited number of participants. The limitations of pharmacological research in elephants must be considered and justify the analysis of clinical cases [22]. With the current North American managed population at approximately 50 breeding females, gathering large study data of a drug such as altrenogest, with a specific purpose in pregnant females, is not feasible [22].

Reproduction in managed elephants has been a growing concern for over 20 years. The facility in which these cases originated has had a history of successful births (>28 births), but the cases described above were not considered normal pregnancies [2,23]. It is unknown why these particular females, two of which previously have had successful births, suddenly exhibited a premature drop in progesterone to baseline levels before full-term pregnancy. The specific reasons each of the pregnant females experienced a premature drop in endogenous progesterone is beyond the scope of this manuscript. Although it was demonstrated that altrenogest was able to maintain pregnancy in Asian elephants (live fetuses confirmed via ultrasonography), it ultimately resulted in unsuccessful births. Administration of altrenogest to other nondomestic species during pregnancy has resulted in varying degrees of successful live births [24,25,26,27,28,29]. Treatment of an okapi *(Okapi johnstoni*) with confirmed progesterone insufficiency and a history of multiple abortions resulted in a live, full-term calf [28]. Likewise, administration to a Sumatran rhinoceros (*Diceros bicornis*) with a history of multiple abortions resulted in a live calf, and administration to a black rhinoceros (*Diceros bicornis*) over four pregnancies resulted in two live, full-term calves [26,29]. Varying degrees of successful live births have also been seen in bottlenose dolphins (*Tursiops truncatus*) administered altrenogest with or without exogenous progesterone [24]. It should be noted that fetal death can occur for reasons other than deficiency of required progesterones and that some of the pregnancies where the mothers were administered altrenogest in these other species also resulted in stillbirths. Thus, perhaps there are other mechanisms that are involved beyond the role of progesterone that are the root of miscarriages or stillbirths, and that even if progesterone supplements are provided to alleviate progesterone deficiency, fetal death may still be inevitable.

Female elephants are polyestrous exhibiting a 13-to-17-week cycle, composed of an 8–10 week luteal phase and a 4–7 week follicular phase [2,30,31,32]. Paired ovarian follicular waves occur within the inter-luteal phase, with the second wave bearing the dominant follicle that ovulates [32]. The corpus luteum (CL), which forms after ovulation, serves as the initial source of progesterone during pregnancy, however, the CL is unique in elephants in that the primary progestogen secreted by the luteal cells is not progesterone specifically [32,33]. The 5α-reduced pregnane metabolites 5α-pregnane-3,20-dione, 5α-pregnane-3-ol-20-one, and 17α-hydroxyprogesterone are produced by the corpus luteum rather than progesterone [1,2,32,33]. Subsequently, reproductive activity can be monitored by multiple methods, including monitoring progesterone levels via pregnane metabolites in serum, urine, feces, or saliva [1,2,6,7,34].

Elephants have the longest gestation period of any mammal, lasting between 20 and 23 months on average, with a reported range between 631–695 days (reported mean of 656+/−18 days) for Asian elephants [1,2,30,31,35,36,37]. The elephant placenta is chorioallantoic with a zonary endotheliochorial structure [2]. The placenta itself is endocrinologically inert in elephants, as the fetal gonads enlarge during the second half of gestation as they begin to synthesize 5α-dihydroprogesterone and other 5α-pregnane derivatives from cholesterol and pregnenolone [8]. While it is assumed that maternal luteal deficiency resulted in the drop in progestins observed in these cases, further studies are needed to evaluate if abnormalities in fetal progestin production somehow contributed to the stillbirths observed or not. The sudden drop and return to near zero (baseline) levels occurring two to three days prior to parturition in elephants means that if altrenogest had not been administered these pregnancies would have certainly resulted in a miscarriage at an earlier time in gestation [1,6,7,8].

The single dose of 0.02 mg/kg administered to two nonbreeding cows represents pilot data of pharmacokinetic values of altrenogest in managed elephants. The half-life (47.5 ± 4.2 h) as determined in these data is substantially higher for Asian elephants than domestic horses (2.6 h after a single dose) and domestic swine (7.24 ± 0.98 h) [21,38]. Although Asian elephants and horses are both hind-gut fermenting herbivores, elephants have a different form of liver metabolism that results in the production of bile alcohols instead of bile acids [22,39,40,41]. It has been postulated in other elephant pharmacokinetic studies that this difference in liver metabolism can result in enterohepatic cycling of drugs, which occurs by biliary excretion and intestinal reabsorption of solutes and is typically associated with multiple peaks and a longer apparent half-life in concentration time profiles [42,43]. While the specifics of altrenogest metabolism have not been described in Asian elephants, the metabolism of altrenogest in horses is in the liver with secretion as glucuronides and a minor portion that is directly sulfated [20]. Equine metabolism involves a portion of the drug undergoing stereo inversion of the 17-hydroxy group (β→α) followed by glucuronidation with another portion of the drug directly sulfated [20]. If metabolism is similar in elephants, the long half-life identified is likely due to enterohepatic cycling. This may also account for the dramatic increase in serum altrenogest concentrations observed in Case 2 when dosing frequency was increased from once per day to twice per day. The time of parturition after cessation of altrenogest administration in these elephants ranged from 15 to 31 days, which is also longer than domestic horses, but agrees with the longer half-life seen in the pilot study [44].

Case 1 received a lower daily dose than what has been described as appropriate for domestic horses to maintain pregnancy, while the 0.02 mg/kg BID administered to Case 2 and Case 3 is the same as the 0.04 mg/kg once daily dose described for horses [11,16,45]. This twice per day dosing resulted in mean serum concentrations of 86.2 + 33.9 ng/mL in Case 2. This is comparable to the serum concentration in horses, ranging from 23–75 ng/mL after receiving five consecutive doses of 0.04 mg/kg once daily [21]. It is also similar in swine, which demonstrated serum concentrations of 66.16 ± 19.94 ng/mL after multiple doses of altrenogest [38]. It is not possible to definitively determine if once or twice per day dosing is appropriate in elephants from the data obtained in this study.

Further studies, considering differences in drug metabolism, are needed to be able to accurately compare parameters of altrenogest administered to elephants to other species. Altrenogest will never be administered as a single dose in clinical patients to maintain pregnancy, therefore, the maintenance of live fetuses in the pregnant cows receiving altrenogest at a dose of 0.01 to 0.02 mg/kg BID should serve as a starting point for future pharmacokinetic studies.

## 6. Conclusions

Administration of altrenogest at a dose of 0.01 to 0.02 mg/kg BID contributed to the maintenance of pregnancy in three Asian elephants with serum progestin levels that had dropped below baseline (<0.020 ng/mL) levels.

## Figures and Tables

**Table 1:** Summary of samples analyzed from clinical cases. Case 3 did not have serum samples analyzed for altrenogest.

Case Number	Initial Altrenogest Dose	Duration of Initial Dose (Days)	Number of Samples Analyzed	Mean Serum Altrenogest Concentration (ng/mL)	Increased Altrenogest Dose	Duration Higher Dose was Administered (Days)	Number of Samples Analyzed	Mean Serum Altrenogest Concentration (ng/mL)	Duration Altrenogest Was Detectable in Maternal Serum after Final Dose (Days)
Case 1	0.01 mg/kg once per day	20	4	2.7 ± 1.5	0.01 mg/kg twice per day	131	31	13.0 ± 6.9	4
Case 2	0.02 mg/kg once per day	95	15	51 ± 20.7	0.02 mg/kg twice per day	210	26	86.2 ± 33.9	15

**Table 2:** Summary of clinical case pregnancies.

Case Number	Age at Conception	Time of Gestation that Progesterone Decreased to Baseline (<0.20 ng/mL)	Time of Gestation Altrenogest Began	Time of Gestation Altrenogest Stopped	Length of Altrenogest Therapy	Pregnancy Length	Time of Parturition after Stopping Altrenogest
Case 1	14 years	489 days	490 days	621 days	131 days	637 days	16 days
Case 2	~48 years	351 days	351 days	561 days	210 days	592 days	31 days
Case 3	10 years	568 days	568 days	621 days	53 days	636 days	15 days

**Table 3:** Summary of pharmacokinetic parameters for two female Asian elephants administered a single dose of altrenogest orally.

Pharmacokinetic Parameter	Mean	± Standard Deviation
AUCext (%)	11.99	1.6
AUC∞ (ng·h/mL)	635.4	73.8
C_max_ (ng/mL)	30.2	14.4
Clss/F	18.0	2.4
Terminal T½ (h)^2^	47.5	4.2
Disappearance λ (1/h)	0.01	0.001
MRT (h)	36.0	3.4
T_max_ (h)	4	2.8
Vd/F (L/kg)	1243.8	275.0

## Data Availability

The data presented in this study are available in the manuscript.
